# Supplementary figures and images for: Transcriptome profiling of laser-captured crown root primordia reveals new pathways activated during early stages of crown root formation in rice
Source: PLoS One. 2020 Nov 19;15(11):e0238736. doi: 10.1371/journal.pone.0238736 (PMC7676735; doi:10.1371/journal.pone.0238736)

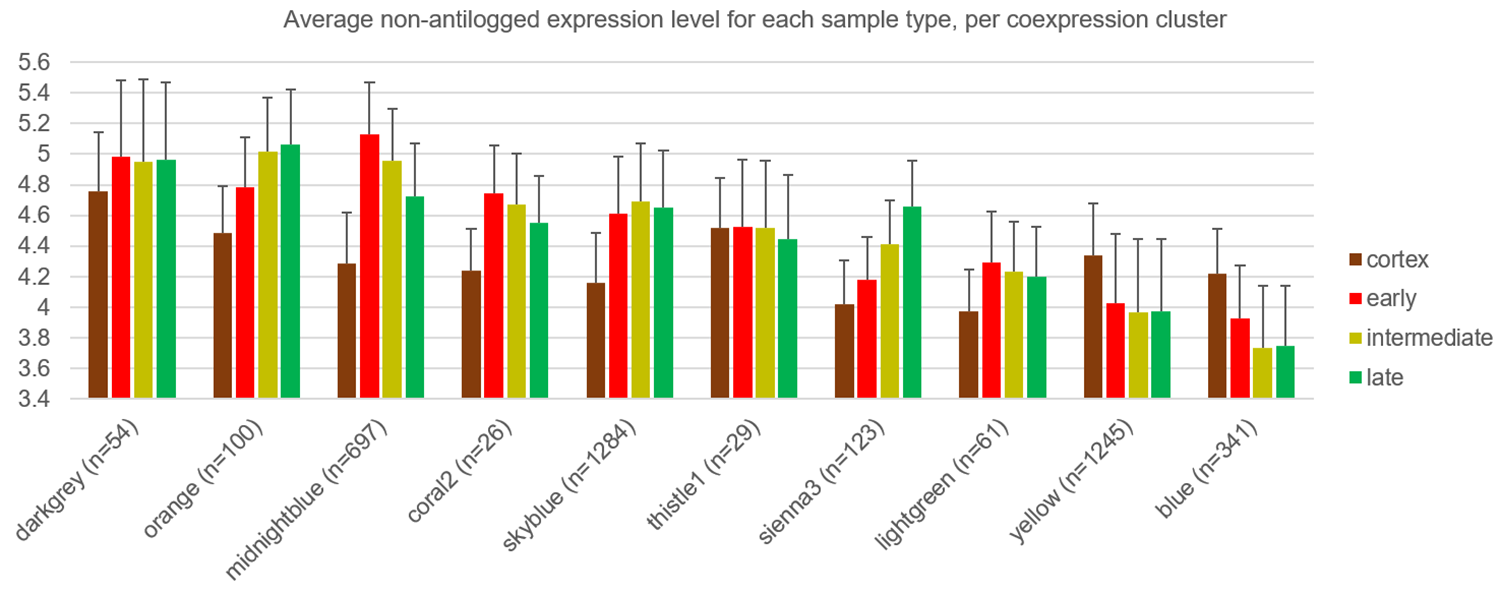

Supplement: S1 Fig — Error bars represent the average relative standard deviation calculated from the three biological replicates. (TIF) [file pone.0238736.s001.tif]

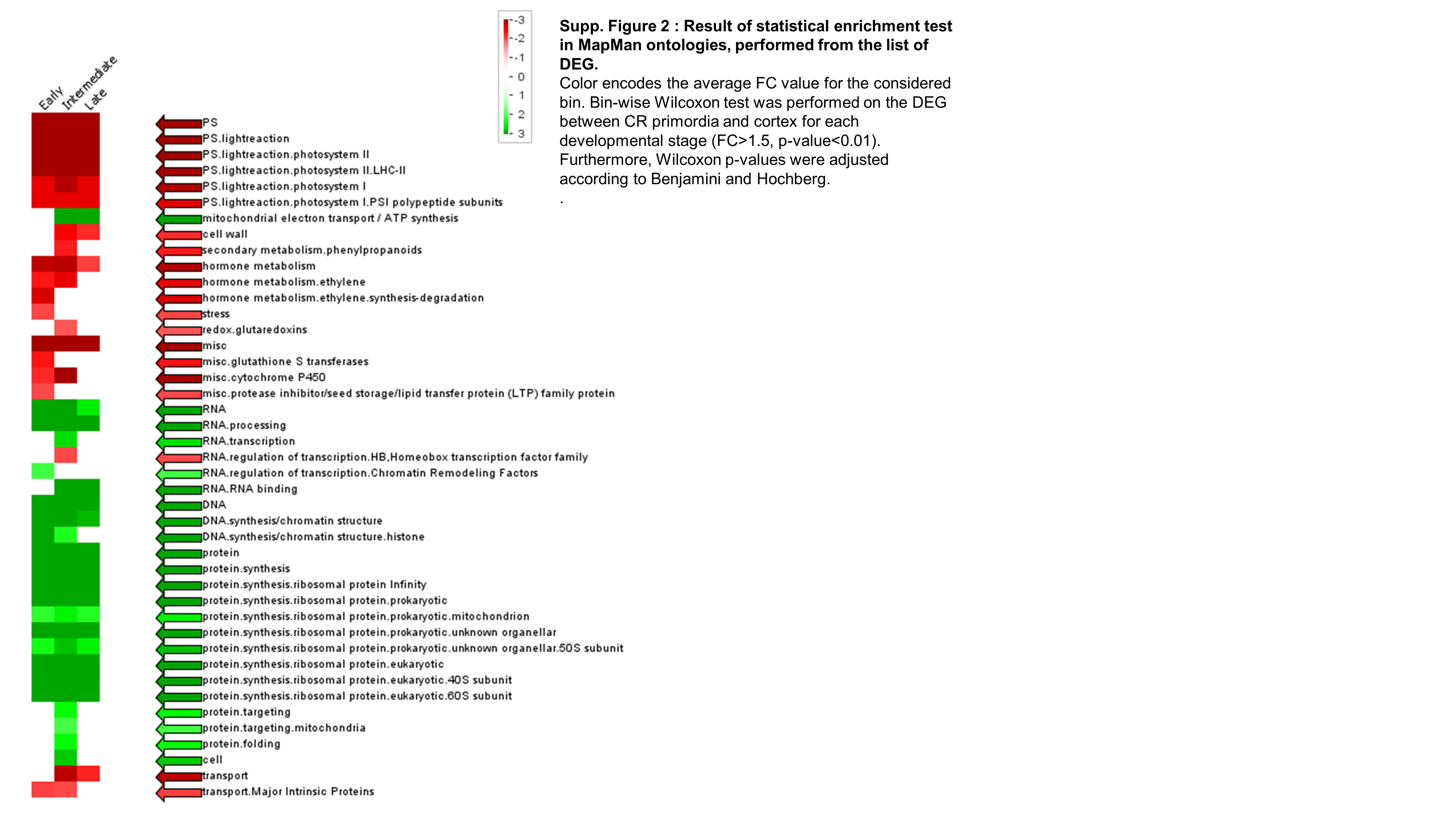

Supplement: S2 Fig — Color encodes the average FC value for the considered ontology. Bin-wise Wilcoxon test was performed on the DEG between CR primordia and cortex for each developmental stage (FC>1.5, p-value<0.01). Furthermore, Wilcoxon p-values were adjusted according to Benjamini and Hochberg. (TIF) [file pone.0238736.s002.tif]

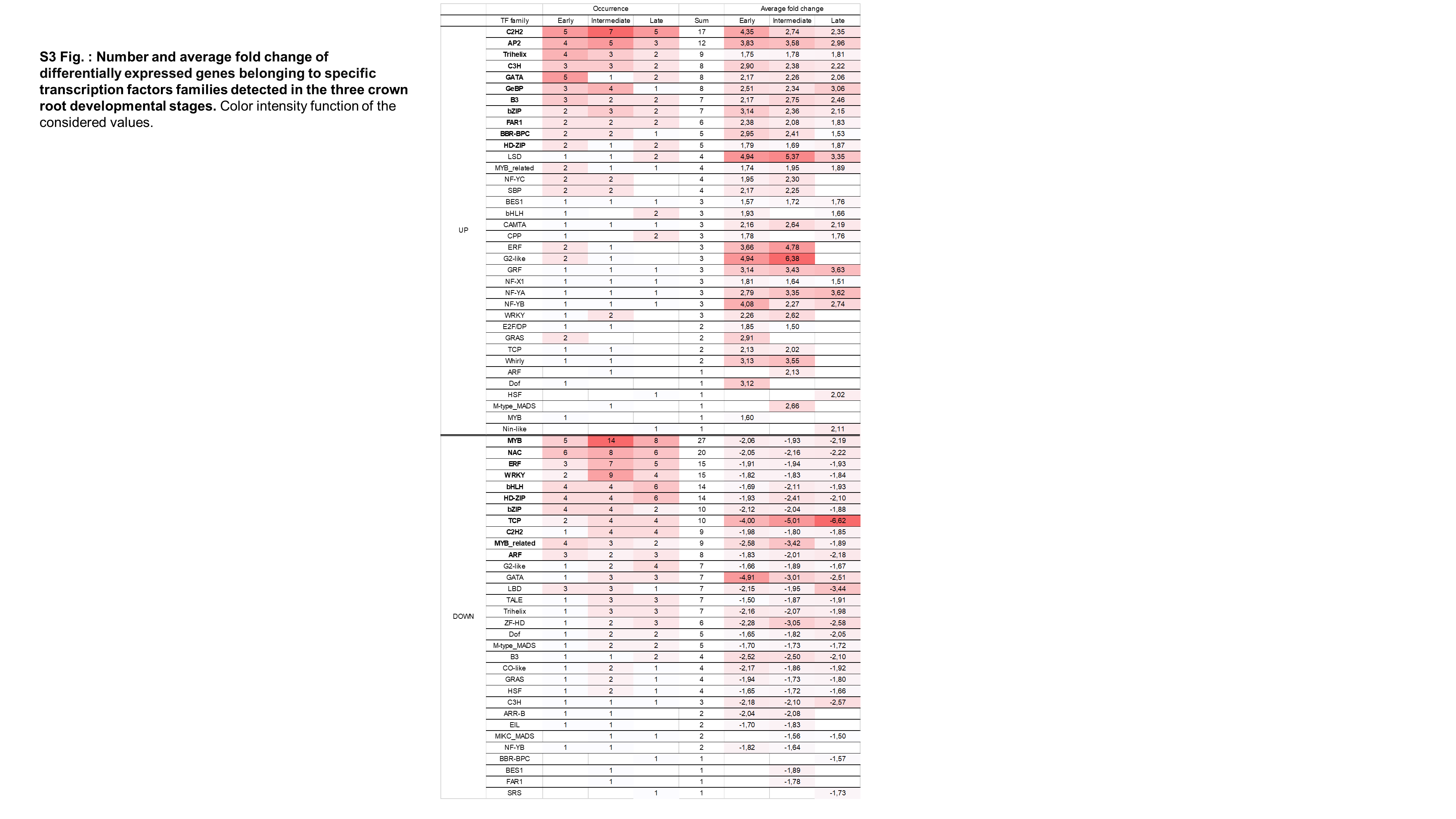

Supplement: S3 Fig — Color intensity function of the considered values. (TIF) [file pone.0238736.s003.tif]
